# A qualitative study on perceptions of surgical careers in Rwanda: A gender-based approach

**DOI:** 10.1371/journal.pone.0197290

**Published:** 2018-05-10

**Authors:** Sojung Yi, Yihan Lin, Grace Kansayisa, Ainhoa Costas-Chavarri

**Affiliations:** 1 Department of Surgery, Centre Hospitalier Universitaire de Kigali, Kigali, Rwanda; 2 Program in Global Surgery and Social Change, Harvard Medical School, Boston, Massachusetts, United States of America; 3 Department of Surgery, Rwanda Military Hospital, Kigali, Rwanda; Indiana University, UNITED STATES

## Abstract

Access to surgical care in low- and middle-income countries (LMICs) remains deficient without an adequate workforce. There is limited understanding of the gender gap in surgical trainees in LMICs. In Rwanda, females fill only one of 20 positions available. Understanding surgeons’ experiences and perceptions of surgical careers may help facilitate support for females to contribute to the global surgical workforce. We performed qualitative analysis on perceptions of surgical careers through semi-structured interviews of all female surgeons (n = 6) and corresponding male surgeons (n = 6) who are training or have trained at University of Rwanda. Transcripts were analyzed with code structure formed through an integrated approach. Question categories formed the deductive framework, while theoretical saturation was reached through inductive grounded theory. Themes were organized within two key points of the career timeline. First, for developing interest in surgery, three main themes were identified: role models, patient case encounters, and exposure to surgery. Second, for selecting and sustaining surgical careers, four main themes emerged: social expectations about roles within the family, physical and mental challenges, professional and personal support, and finances. All female surgeons emphasized gender assumptions and surgical working culture as obstacles, with a corresponding strong sense of self-confidence and internal motivation that drew them to select and maintain careers in surgery. Family, time, and physical endurance were cited as persistent challenges for female participants. Our study reveals concepts for further exploration about gendered perceptions of surgical careers. Efforts to improve support for female surgical careers as a strategy for shaping surgical work culture and professional development in Rwanda should be considered. Such strategies may be beneficial for improving the global surgical workforce.

## Introduction

Inadequate workforce is a fundamental challenge to addressing the disproportionate burden of surgical diseases in low- and middle-income countries.[1][2] The Lancet Commission on Global Surgery recommends a density of 20 surgeon, anesthesiologist, and obstetrician (SAO) providers per 100,000 population as a conservative benchmark correlated with improved health outcomes.[3][4] However, in most regions of Africa, there are more than 100-fold fewer surgeons per capita than in the United States or Europe.[5] Rwanda, a low-income country in East Africa,[6] reports approximately 0.8 SAO providers per 100,000 population, with only 51 fully trained surgeons in the country.[7]

Despite the significant need, women remain an untapped resource for expanding the number of surgical professionals worldwide.[8][9] In Rwanda, similar numbers of women and men graduate from medical school, but women continue to be underrepresented in surgery, with an average of one female surgical trainee per 18 males.[7] No medical association or governing entity in Rwanda has limited the number of surgeons, and eligibility is determined through successful examinations similar to the process led by the American Board of Surgery, but adjusted for the Rwandan surgical training needs. An important strategy for increasing the number of trainees in Rwanda, then, is by building interest and reducing barriers for women to pursue surgical careers.

Studies from other African countries have tried to understand why people select and maintain surgical careers. In Zimbabwe, experiences in leadership roles have helped shape female surgeons’ selection of specialty,[10] while surveys of Ethiopian surgeons[11] and Kenyan medical students[12] reveal that preference for location and practice setting contribute to surgical trainee retention and students’ selection of surgical specialisation. These variables are context-specific, as historical underrepresentation of female surgeons in Zimbabwe may affect how current trainees perceive and select their careers, and complex geopolitical forces that have shaped Ethiopia and Kenya’s socioeconomic development may also affect where and what trainees choose to practice. Additionally, a majority of medical students in Nigeria cited concern for the increased risk of HIV/AIDS associated with surgical specialties, though it ultimately did not have a statistically significant effect on specialty selection.[13] Such contextual variables shift differently in low- and middle-income countries, and findings from these studies in the region suggest that there may be factors that are unique to Rwanda as well.

As the Rwandan health care and medical education systems continue to grow rapidly, it is important to understand why women are less likely than men to choose careers in surgery, and especially how the current Rwandan female surgeons have experienced this process so far. Our study examines the experiences of both female surgeons and their male counterparts in Rwanda to provide a window into critical decision points that shape career paths. We believe that understanding the experiences and perceptions of surgical careers amongst female and male surgeons may help improve policies and programs for supporting women to contribute to the global surgical workforce.

## Materials and methods

This study is a cross-sectional qualitative survey of all female surgeons in Rwanda and their corresponding male surgeon colleagues. An integrated approach was used to explore the contextual variables involved in selecting and building a surgical career in Rwanda: a deductive organizing framework was built for the interview guide, and an inductive, constructivist grounded theory approach was applied to the data to develop new theoretical constructs from the “ground up”[14]. We adhered to the consolidated criteria for reporting qualitative research (COREQ).[15]

### Study setting

Rwanda is a low-income country in East Africa,[6] where tremendous recent investment in health care has improved life expectancy and infant/maternal health indicators.[16] In 2012, the Rwandan Ministry of Health partnered with a consortium of US academic institutions to create the Human Resources for Health (HRH) Program to increase the quantity and quality of providers in Rwanda.[17] The number of surgical residents increased from 15 in 2012 to 50 in 2016. Despite such progress, significant challenges in infrastructure, staff, and clinical processes remain for delivering safe, effective, and timely surgical care.[18] It is in this context that Rwanda provides an ideal background for understanding perceptions about entering and building surgical careers amidst growing yet still resource-limited settings.

### Participant selection

Using a purposive sampling technique, we interviewed all female surgeons who had been trained in Rwanda (n = 6), as well as male surgeons at corresponding career points (n = 6), for a total of 12 surgeons. After matching the number of years in training or practice to their female counterparts, male surgeons were selected randomly from a list of providers through the Department of Surgery at the University of Rwanda. Of note, there are female and male surgeons in Rwanda who have trained in other countries or work temporarily in Rwanda; however, our objective was to understand the perceptions of those who had trained in Rwanda. All participants were approached in person or via mobile phone and provided informed oral consent to take part in the study. Participants were also offered a written statement describing the project and their rights to withdraw themselves from the study. No individuals approached declined to participate.

### Researcher team

The research team included a surgeon, two surgical residents, and one medical student- all of whom are female. The primary interviewer (SY) is a US medical student with experience in qualitative methodology. The data collection and analysis process were managed by SY and a US surgical resident with training and experience in qualitative methodology (YL). The surgeon (ACC) and surgical resident (GK) who have been living and working in Rwanda provided counsel with contextual insight during the analysis phase, as GK participated in the study as well. Prior to the study, SY worked in Rwanda for 5 months and met with Rwandan surgical residents and surgeons to explain the goals of the study.

### Data collection

The interviews were semi-structured with evolving open-ended questions.[19] Our interview guide consisted of questions on the chronological process of selecting and building a career in surgery. This structure enabled the interviewer to collect information about how participants became interested in surgery, how they perceive the specialty, and which aspects continue to motivate them, while still giving informants the opportunity to report on their own thoughts, gender assumptions, and feelings. Two pilot interviews were conducted on a surgeon and surgical resident in Rwanda, but only minimal changes for clarifying the wording in the interview guide were made. Interviews were conducted in private and performed in English. No repeat interviews were conducted. Interviews and informed oral consent were audio-taped when permission was given, then transcribed verbatim. Per the IRB protocol approved by the University of Rwanda College of Medicine and Health Sciences, all electronic recordings and documents were appropriately secured to preserve participants’ desired anonymity. All interviews were conducted from December 2016 to January 2017.

### Data analysis

Analysis occurred in two phases. In the first phase, two members of the research team (SY, YL) read the transcripts and inductively created a code structure. Transcripts were assigned in ATLAS.ti (version 1.01.50; Scientific Software Development GmbH, Berlin), and open coding was used to assign and generate themes and subthemes for a final code structure. The categories of questions from the interview guide represented key conceptual domains for the deductive framework. An inductive grounded theory approach was then taken to develop the codes and code structure until the point of theoretical saturation,[20] which was defined as the point at which no new concepts emerge from reviewing of successive data from a diverse sample set. Theoretical saturation was reached after four interviews. Two researchers independently applied the codes from the finalized code structure, reviewed discrepancies, and resolved differences through in-depth discussion and negotiated consensus.

In the second phase of analysis, the coded transcript was explored to describe and form relationships within the content. The results provided insight into the nature of selecting specialties, as well as participants’ perceptions about gender-based roles and their influence on surgical careers.

### Member checking

Contextual validity was sought through sharing preliminary study findings to Rwandan surgeons and residents so that they could respond to and advise on the validity of the emerging themes. The process of “member checking” facilitated a feedback mechanism on the interpretation of our results from individuals who are living and working daily in the context of this study.[21]

### Institutional approval

Ethical approval for the study was obtained from the Institutional Review Board at the University of Rwanda College of Medicine and Health Sciences: Approval Notice No. 362/CMHSIRB/2016.

### Ethical considerations

The informed oral consent procedure was reviewed and approved by the Institutional Review Board at the University of Rwanda College of Medicine and Health Sciences. Consent for participation was recorded, and no subjects refused participation. Written consent from the participants was not pursued or stipulated by the Institutional Review Board in order to minimize the physical supervision and security needed to store identifying information.

Limited data from this study is only available upon request, as there are ethical restrictions on sharing the de-identified data set publicly. First, the interview transcriptions are inextricably linked to the participants, as the content reflects their experience training in Rwanda so far and cannot be de-identified appropriately without removing most of the data. Second, the Institutional Review Board approved a data collection, security, and storage procedure in which the original transcriptions must be erased and destroyed upon completion of the written work for this research. The contact point for accessing the de-identified data is the corresponding author.

## Results

A total of 12 surgeons were interviewed: six females and six males. One-third of interviewees (n = 4) are attending surgeons, as there are only two female attending surgeons in Rwanda, and their two male counterparts were interviewed. The remaining two-thirds of interviewees (n = 8) are surgical residents training in post-graduate year PGY-1, PGY-2, PGY-3, and PGY-5. All 12 surgeons indicated that they are already practicing or intend to practice surgery. At the time of the study, twice as many male than female participants reported being married, and an equal number of male and female participants reported having children. Interviews were conducted over a four-week period, and each interview lasted an average of 42 minutes, ranging from 33 to 56 minutes.

We identified main themes based on two defining points in the participants’ career timeline: developing interest in surgery as a specialty, and selecting and sustaining surgical careers (S1 Table). Certain interview questions asked explicitly for participants’ opinions about gender-based reasons for differences in approaching surgical careers, but responses to other questions also revealed assumptions about gender roles in Rwanda. Each theme and subtheme were thus analyzed through a gendered lens.

### Developing interest in surgery as a specialty

When asked to identify key experiences that introduced surgery as a possible specialty to consider, participants described three main themes: 1) role models, 2) patient case encounters, and 3) exposure to surgery. Regardless of gender, identifying a surgeon role model who demonstrated the feasibility of pursuing a surgical career was important for most participants. Women appeared to emphasize interpersonal experiences with patients and other surgeons as motivating their interest in surgery. In contrast, men pointed more to results seen from positive hands-on experiences, especially ones where their surgical skills were singled out and complimented.

#### Role models

Most participants reported that observing surgeons was important to piquing their interest in surgery as a specialty. This opportunity to observe and gain hands-on experience in the operating room is built-in as a required clinical rotation for all medical students in Rwanda. All female participants defined important role models as each other, as well as select male colleagues who encouraged them. One female surgeon recalled, “it was important that [she] was there… At that time, I didn’t know that I could do it too. That alone couldn’t have helped me decide, but [she] really made me say, let me try” (F5). The male figures commonly cited by the female participants as “encouraging” qualified by promoting the idea that females could also train in surgery despite it being a male-dominated field. Multiple female surgeons recalled that even this initial act of “thinking about it, considering the career” (F6) involved a role model who demonstrated or verbally supported the feasibility of this career path for women.

In contrast, male participants cited attending surgeons who expressed confidence in the trainees’ technical ability to succeed. Many participants mentioned having foreign surgeons “find” them (M2). A role model often acknowledged an innate talent for a surgical skill that then motivated the male participant to develop an interest in surgery: “good at tying”(M4), “good at cesarean sections”(M5), “good at anastomoses”(M6).

#### Patient encounters

The importance of interpersonal influences on female participants was also apparent in their descriptions of surgical patients. All females shared some critical aspect of surgical patient care that influenced their selection of surgical careers. Some described being drawn to the brief intensity of caring for surgical patients, because there is an operation and solution to offer, instead of the often intangible or long-term care needed in other specialties. As students, they could observe the immediate benefits of surgery on their patients, in contrast to the slow or lack of visible improvement of patients on their brief pediatrics or internal medicine rotations. Being able to provide a tangible service engendered a sense of accomplishment and affinity for surgery.

Alleviating suffering for patients was another appealing aspect of surgery for some females. Limited pain relief options for children or for mothers during childbirth appeared to have left negative impressions. Surgery, with its anesthesia and lasting potential benefits, represented a type of patient care that could be done without pain to alleviate pain. Not being able to manage the suffering of their patients evoked feelings such as “fear,” “shame,” “discomfort,” and “not be satisfied” in these female participants (F2, F3, F4, F5)

In contrast, no male participants mentioned the impact of their care on patients as factoring into their decision for selecting surgery. Instead, many described the appeal of patient care from an intellectual perspective, using terms such as “straightforward,” “straight,” or “clear” to describe the clinical pathways involved in patient cases. Only one female cited this intellectual clarity as a significant factor in her decision. A male participant explained, “I like surgery because it is clear… you make a diagnosis and then there is a clear plan and then you can do it. This is different than internal medicine, where you may have a patient, not know the diagnosis. And even if you have diagnosis… you can have mixed results” (M1). Rather than focusing on how they may feel from personally affecting patients, many male participants emphasized how they felt from the clinical thinking process stimulated by patient cases.

#### Exposure to surgery

Participants reported exposure to surgery through rotations during medical school, or while working as general practitioners in rural district hospitals. Only two female participants explicitly cited the hands-on experience of operating as a factor in their interest in surgery: “I think I have good hands-on skills [to do surgery]” (F1), “I like being in the OR just operating, doing something that you see the result of. I can wash out an abdomen, do a resection, make anastomoses, and the patient goes out. You are satisfied” (F3).

All male participants cited exposure as a main factor in the development of their interest. They offered other reasons as well, but the time spent in the operating room directly observing the surgeries appeared to help solidify their decision to enter the specialty. One surgeon emphasized this experiential bias: “Everyone had something that happened to them to select their specialty. I changed my mind during my 5th year… It came abruptly, [after] seeing cases during rotations” (M2). Another surgeon described regular interaction with international surgeons who were “helping people in the district, and I saw that it [surgery] was good. I started operating on patients too, doing hernias… And this is when I chose” (M5). While this participant alluded to the beneficial impact of surgeries on helping people at the district hospital, he continues on to clarify, “even minor surgeries, excising lipomas, cysts…it was just from there that I starting liking surgery,” shifting the focus from patient care to the operative exposure.

### Selecting and sustaining surgical careers

After observing the main factors that initiate interest in surgery, we sought to understand how surgeons then navigated their process for ultimately selecting and training in surgery as their specialty.

Four themes emerged surrounding 1) social expectations about roles within the family, 2) physical and mental challenges, 3) professional and personal support, and 4) finances. Representative quotes are included in the following section and S2 Table.

#### Social expectations about roles within the family

Gender assumptions played a significant part in participants’ perceptions about building careers and particularly careers in surgery. All participants acknowledged that “surgery takes time” (F5), with each gender citing different competing interests for their time.

For the female surgeons, expectations about what it means to inhabit a female role within the family or societal structure often felt at odds with the demands of a surgical career. Females discussed social challenges of getting married or, if they were already married, having children. These concerns were described in terms of the social pressure that women should fulfill certain roles, and the physical accommodations during pregnancy and child-rearing. One surgeon summarized this conflict as: “When you want to join surgery, you know how demanding it is, but if you like it, you tend to sacrifice other things. You can choose another [specialty] for the sake of family and other social responsibilities” (F6). They observed, however, that they alone could not overcome this gender stereotype, as “the fact is marriage has a very big effect. Even if there are some who have [an] open mind, there are very few [and] they cannot determine the society” (F3). They insisted that it would take conscientious effort from both male and other female colleagues to address these biases.

Male participants offered mixed views on social responsibilities, with some volunteering that limited time with their families may present a challenge, while some stating that they do not anticipate any personal concerns for their surgical career. One male surgeon responded that “I’ve managed to balance the two things: too much work load but also having time with social pattern. I have never abandoned my social life” (M1). Another male participant framed the gender expectation as “time for your family is not the primary thing that men think about, but for women they would think about that before anything else” (M6).

#### Physical and mental challenges

In addition to these social pressures, the physical demands of surgery were framed in terms of gender expectations. All female participants recalled physical stereotypes, such as “women are less strong than men” (F4), being imposed upon them. They all defied these assumptions, acknowledging the physical challenges of the work but refusing to concede that being female must be a limitation. One surgeon’s statement captured this attitude: “Women may be physically, emotionally less strong than men. But me, I am not that way, because I feel that I am strong as men” (F4).

Moreover, some females reported that there were limitations in mindsets within personal relationships, where male figures may seek to exert control over female activities. This control was described both in marital relationships and at a societal level: “We are in Africa. [The husbands] say you can do this, you cannot do this” (F3), and “in Rwanda, they say there are some jobs that are made for males and other jobs made for women, culturally… In sciences, people think it was made for males not for females” (F3). Even after overcoming such gender expectations to become a surgeon, one female participant shared that “patients still hesitate a bit with me and other people ask me, why [surgery]? And I say to them, 99% of [surgeons] are male. Do you ask them the same question?” (F1).

In the process of overcoming this mentality about not belonging, female participants often mentioned fear in terms of self-doubt as a possible factor contributing to the gender divide in surgeons. Only female participants mentioned feeling personally responsible for the outcome of their patients as a significant challenge of surgical practice. A negative outcome, such as complications from surgery or even mortality, was internalized, expressed in phrases such as “I feel responsible” (F3), or “maybe the patient has even died because of me” (F4). One surgeon explained, “Some women just can’t make it to surgery. People got scared in undergraduate; they showed us that surgery is like a fight” (F3). Another even drew on biological roots, with “testosterone. Men do not fear, and female do fear. So, when in surgery, gynecology, anesthesia, you need a high level of androgen to do it, self-confidence” (F1). In response to this perceived need for fearlessness in surgery, female participants rejected the gender stereotype: “I am judged, but not because I’m a woman, but because I’m like everyone else. I don’t consider myself as female” (F6). Others shared their experiences of overcoming fear through perseverance, “I think I am quite persistent with what I do. I will not really fail to do something" (F5).

When asked why more men enter surgery than women in Rwanda, male participants speculated that it was female mindsets that shaped their preference, rather than any systemic gender-based bias. One surgeon summarized this difference in genders: “surgery requires brain, requires physical work, [and] working hard. Males… in our culture and all over the world, they are stronger so they have been doing stronger things” (M2). Instead of recognizing this gender assumption, many male surgeons placed the onus on their female colleagues: “There really is no sex selection criteria for doing surgery. It is all ‘mentality-driven’” (M1), “factors that push women away from surgery are in their minds” (M2), “what a man can do, a woman can do it” (M3), “women don’t like blood” (M6). Beyond this mental barrier, male participants described that females may not feel fit for the physical demands of surgical careers. While this preference is admissible for any provider, the descriptions were contextualized in terms of gender: “[women] think that job for women should be easier, that they don’t have to work hard” (M4), and “females do not like stress. They like easy life, like pediatrics, radiology, something in which they are not stressed and can just sit down” (M4).

Despite the demands and risks of surgical careers, the hands-on nature of operating seemed to sustain all participants, as they described how rewarding it felt for their work to translate into outcomes for patients. All interviewees cited the importance of developing a tactile skillset of operating. One surgeon explained, “That is the special thing about surgery … You can touch it, it is palpable … you can do it, you can do you right things. You can expect your outcomes” (M1). Another compared surgery to other specialties such as “internal medicine: they work like a witch! You just give many, many medications, you never know which one is treating the patient, which one is killing him… In surgery, you always know what is happening” (F5).

#### Professional and personal support

All surgeons also described the difficulty of working in an environment that lacks the proper infrastructure, materials, equipment, and surgical staff to learn and operate effectively. Also, given limited opportunities for further training in Rwanda, some surgeons, both male and female, remained hopeful that pursuing surgical careers could lead to more international opportunities.

Navigating the work place includes negotiating relationships with peers, mentors, and institutional hierarchies. All surgeons regardless of gender described challenges in managing expectations of others and seeking positive learning experiences. All participants also described a professional mentor in the form of an attending surgeon or surgical resident who had been critical to supporting their careers. The definition of what this professional support looks like varied widely, from technical guidance and responding to questions during operations to spiritual counsel and motivation during difficult cases. All females described the importance of “respect” and “independence” in both personal and mentor relationships, which were not mentioned explicitly by males. A few females further clarified independence as feeling they were making decisions and operating without an overbearing presence. Only female participants shared an explicit desire to support and mentor future generations.

Most male and female surgeons appeared to find support from their colleagues, their nuclear families, and their own families.

#### Finances

All males cited inadequate financial compensation as their main concern for sustaining surgical careers. They explained that financial restructuring would need to occur at a larger institutional or national level to retain surgeons and to offer higher-level training. Many surgeons described their concern for the mismatch in compensation and time: “when I see consultants (surgery, anesthesiology) leave, I begin to worry. You train someone for that long, for 10 years, you should be training others. But they leave for themselves. [they] need money, and they leave for NGOs, private” (M5), and “when you spend 15 years in training, at some point you need something back, and that thing should be money” (M6).

In contrast, most females mentioned financial stability and job prospects as advantages of surgical careers, because there are “no concerns about job prospects. We have like 100 years of need for surgeons in Rwanda. The future is bright; I have no concern about financial prospects” (F5).

## Discussion

Factors that motivate interest in surgery are highly interpersonal for the Rwandan female participants. Prior studies reveal that students with surgical role models are more likely both to aspire to a surgical career and to report an understanding of the demands of such a career.[22] Despite few female surgeons to support trainees’ reconciliation of surgical and gender roles, female Rwandan surgeons reported that both male and female role models have been crucial to encouraging the possibility of simultaneously attaining social responsibilities, professional aspirations, and even physical fitness to pursue a surgical career. Moreover, only the women in this study cited an aspect of surgical patient care that appealed to their desire to relieve suffering and help patients in a tangible way. The benefits of such empathetic perspectives on patient care can be invaluable. Literature has shown that female physicians use more patient-centered communication,[23] provide more psychosocial counseling to their patients,[24] and even produce lower mortality and readmissions compared to male physicians.[25] The emphasis on interpersonal connections between surgeons and patients by our study’s female participants suggest that there may be differences in motivation and perhaps practice patterns with important clinical implications for patient care.

While female responses framed the value for surgery’s direct impact in terms of the effect on their patients, male participants expressed this value in clinical terms. Many men described developing an interest in surgery because of intellectual clarity in surgical pathways, where perhaps pathologies and treatment options appeared to be matched. Whereas female participants cited role models for helping overcome social barriers to considering surgical careers, male participants explained developing interest in surgery after having technical skills noticed and commended by surgeons. These concerns that the Rwandan male surgeons expressed echo other analyses of gendered experiences in workplaces and within medical learning environments.[26] One study found that stereotypically male attributes of physical strength and competitiveness[27] were valued within surgery and led female students to identify less with that career. Indeed, our Rwandan female surgeons spoke of the physicality and time required to enter and compete in a surgical career, while their male counterparts highlighted the clinical and technical appeal, implying that such reservations did not significantly influence their career decision calculus.

To sustain their surgical careers, female participants again described a strong bias against surgery based on social expectations about their gender role in Rwanda and the physical stereotypes of women. However, each discovered a way to reconcile these prevailing perceptions with their career. They described and promptly rejected the fear that arose from the pressure to address these gender-based biases, with some even shedding gender altogether (“I don’t consider myself female”). This rejection of femininity and careful re-presentation of oneself reveals an opportunity for organizational structural support to address gender biases in Rwandan medical culture and for thoughtful leadership development to encourage gender diversity. Social expectations about the male role in Rwanda appeared to revolve around the pressure to provide financially for the family structure, which became apparent in the unanimous concern for financial compensation. While female participants demanded respect as surgical trainees, surgeons, and members of the larger social fabric, men were more likely to accept the conditions of work environment and instead demand improved financial support. Gender disparities in concern for income may contribute to the increasing portion of female physicians, a process called “feminization of the medical workforce.”[28] Evidence from high-income countries show that female physicians work fewer hours, see fewer patients,[29] and get paid less than their male colleagues.[30] Feminization in medicine has been described in South Africa,[31] but its implications for local human resources, greater healthcare delivery, and ultimately patient care in other low- and middle-income countries has not been studied yet. The divergence in gender experiences in Rwanda calls for larger discussions on empowering women throughout society as countries pursue socioeconomic development.

Concern for low numbers of female surgeons is well-documented in the literature worldwide, with similar issues related to work environment, family, and lifestyle cited by previous studies.[32][33] Women already in surgical careers, however, tend to report satisfactory control over their life-work balance,[34] revealing a discordance between perception and reality of practice, and suggesting a miscommunication within the education structure.[32][35] Likewise, in Rwanda, measures to address and limit the discordance in gender-based perceptions about surgical careers versus their realities are needed as female participants universally report “no regrets” for selecting a surgical career. Institutions have a critical part in supporting women in surgery, as they can not only engineer a “hidden curriculum” where culture, beliefs, and behaviors of the surgeons help shape students’ experiences early on,[36] but also consider formal initiatives to prepare surgical trainees to deal with the realities of their careers and to promote a sense of control over perceived challenges. These programs would foster opportunities where faculty and trainees can informally and formally discuss career concerns, and help improve female inclusion and reject imposed gender-based limitations.

Moreover, prior studies demonstrate empowered mentoring as a key factor in career support and success.[37][38] In our study, all participants regardless of gender cited the importance of having mentors with whom they could consult professionally and personally. Mentorship also looks different for each gender, as these relationships often form when the junior and senior have common experiences.[39] Male physicians may have less success with female trainees, especially as they may withdraw as mentors when female mentees exhibit independence or appear self-sufficient,[40] both traits mentioned by our study’s female participants. Mentors and faculty members must recognize the female mentee tendency to underestimate or hesitate asserting agency within the context of gender stereotypes.[41] In contexts where female surgeon mentors may be scarce, mentorship can be improved by establishing networks for connecting female faculty with regional and international surgical women’s groups. With such gender-mindful mentorship, “sponsorship” also is a strategy for investing in nascent talent including women: those in positions of power can help accelerate the advancement of female colleagues in the workplace by advocating publicly for them.[42] Empowered mentorship, or sponsorship, thus can enhance a trainee’s development in many domains including the personal, professional, and public.

### Limitations

This study is a unique exploration into the challenges facing the surgical workforce in East Africa, though the gender assumptions that emerged may be specific to Rwanda and results not generalizable to other settings. However, given the gender-based challenges echoed in the region and worldwide, and the fact that these interviews included all female surgeons in Rwanda, we believe that the results of this study may represent a diverse array of contexts.

Trustworthiness of the responses was sought by working closely with surgeons and surgical residents who are familiar with the Rwandan context. To reduce the risk that analyses could have been misinterpreted, we performed ‘member checking’ by discussing findings with Rwandan male and female surgeons, who confirmed the accuracy of the data and their interpretations. The similarity of themes across participants further offers support for content validity.

Bias could have been introduced by data coding and analysis, however standardized data collection was performed, with dual coding of transcripts and iterative rounds of analysis.

### Future considerations

Based on the findings of this study, future directions for further investigation would likely include exploring strategies for medical institutions to create supportive programs for women surgeons, address systemic changes to facilitate work-life balance, encourage social networking opportunities for female surgeons.

## Conclusions

Feminization of the medical student profile has prompted research on the global growth of women in medicine,[43][44] and this relatively recent phenomenon illuminates social issues about female and male roles in society. There is a gender-based misbalance in human health resources, and there must be concerted effort to change policies to attract and retain surgical trainees especially in contexts such as Rwanda where a lack of medical specialists has been described.

## Supporting information

S1 Table Main themes. (PDF)

S2 Table Additional example quotes about selecting and sustaining surgical careers. (PDF)

S1 Appendix Sample interview question guide. (PDF)

## References

[1] Myles PS, Haller G. Global distribution of access to surgical services. Lancet 2010;376(9746):1027–8. doi: 10.1016/S0140-6736(10)60520-X 20598364 10.1016/S0140-6736(10)60520-X

[2] Hoyler M, Finlayson SR, McClain CD, Meara JG, Hagander L. Shortage of doctors, shortage of data: a review of the global surgery, obstetrics, and anesthesia workforce literature. World J Surg 2014;38(2):269–80. doi: 10.1007/s00268-013-2324-y 24218153 10.1007/s00268-013-2324-y

[3] Meara JG, Leather AJM, Hagander L, Alkire BC, Alonso N, Ameh EA, et al Global Surgery 2030: evidence and solutions for achieving health, welfare, and economic development. The Lancet Commission of Global Surgery 2015;386(9993):569–624. 10.1016/S0140-6736(15)60160-X 25924834

[4] Daniels K, Riesel JN, Meara JG. The scale-up of the surgical workforce. Lancet 2015;385(Suppl 2):S41. 10.1016/S0140-6736(15)60836-4 26313090

[5] Ozgediz D, Kijjambu S, Galukande M, Dubowitz G, Mabweijano J, Mijumbi C, et al Africa’s neglected surgical workforce crisis. Lancet 2008;371:627–628 doi: 10.1016/S0140-6736(08)60279-2 18295007 10.1016/S0140-6736(08)60279-2

[6] The World Bank. Rwanda: Country at a glance [The World Bank web site]. Available at: http://www.worldbank.org/en/country/rwanda. Accessed April 23, 2017.

[7] Rickard J, Ssebuufu R, Kyamanywa, Ntakiyiruta G. Scaling up a Surgical Residency Program in Rwanda. East Central Africa J Surg 2016;21(1):12–23.

[8] Neumayer L, Kaiser S, Anderson K, Barney L, Curet M, Jacobs D, et al Perceptions of women medical students and their influence on career choice. Am J Surg 2002;183:146–50. 11918878 10.1016/s0002-9610(01)00863-7

[9] Wendel TM, Godless CV, Pinz RA. Are there gender differences in choosing a surgical career. Surgery 2003;134(4):591–96;discussion 596–8. doi: 10.1016/S0039 14605619 10.1016/s0039-6060(03)00304-0

[10] Muchemwa FC, Erzingatsian K. Women in surgery: factors deterring women from being surgeons in Zimbabwe. East Central African J of Surgery 2014;(19)2:5–11.

[11] Derbew M, Laytin AD, Dicker RA. The surgical workforce shortage and successes in retaining surgical trainees in Ethiopia: a professional survey. Human Resources for Health 2016;14(Suppl 1):29 doi: 10.1186/s12960-016-0126-7 27380899 10.1186/s12960-016-0126-7 PMC4943482

[12] Dossajee H, Obonyo N, Ahmed SM. Career preferences of final year medicalstudents at a medical school in Kenya—A cross sectional study. BMC Med Educ.2016 1 11;16:5 doi: 10.1186/s12909-016-0528-1 26754206 10.1186/s12909-016-0528-1 PMC4709906

[13] Ocheke AN, Musa J, Ekwempu CC. The impact of HIV/AIDS epidemic on the choiceof specialties among medical students and house officers in Jos, Nigeria. Niger JMed. 2008 Apr-Jun;17(2):201–4. 18686840 10.4314/njm.v17i2.37384

[14] Miles MB, Huberman M. Qualitative Data Analysis: A Sourcebook of New Methods. 2^nd^ edition Beverly Hills, CA: Sage Publications; 1994.

[15] Tong A, Sainsbury P, Craig J. Consolidated criteria for reporting qualitative research (COREQ): a 32-item checklist for interviews and focus groups. Int J Qual Health Care. 2007;19(6):349–57. doi: 10.1093/intqhc/mzm042 17872937 10.1093/intqhc/mzm042

[16] National Institute of Statistics of Rwanda (NISR) [Rwanda], Ministry of Health (MOH) [Rwanda], and ICF International. 2015 Rwanda Demographic and Health Survey 2014–15: Key Indicators. Rockville, Maryland, USA: NISR, MOH, and ICF International.

[17] Binagwaho A, Farmer PE, Nsanzimana S, Karema C, Gasana M, de Dieu NJ, et al Rwanda 20 years on: investing in life. Lancet 2014;384(9940):371–375. doi: 10.1016/S0140-6736(14)60574-2 24703831 10.1016/S0140-6736(14)60574-2 PMC4151975

[18] Petroze RT, Nzayisenga A, Rusanganwa V, Ntakiyiruta G, Calland JF. Comprehensive national analysis of emergency and essential surgical capacity in Rwanda. British Journal of Surgery 2012;99(3):436–443. doi: 10.1002/bjs.7816 22237597 10.1002/bjs.7816 PMC10767725

[19] Dicicco-Bloom B, Crabtree BF. The qualitative research interview. Med Educ 2006;40(4):314–21. doi: 10.1111/j.1365-2929.2006.02418.x 16573666 10.1111/j.1365-2929.2006.02418.x

[20] Patton MQ. Qualitative Research and Evaluation Methods, 3^rd^ edition Thousand Oaks, CA: Sage Publications; 2002.

[21] Creswell JW, Miller DL. Determining validity in qualitative inquiry. Theory Into Pract 2000;39:124–130.

[22] Ravindra P, Fitzgerald JEF. Defining surgical role models and their influence on career choice. *World J Surg* 2011;35:704–9. doi: 10.1007/s00268-011-0983-0 21312036 10.1007/s00268-011-0983-0

[23] Roter DL, Hall JA. Physician gender and patient-centered communication: a critical review of empirical research. Annu Rev Public Health 2004;25:497–519. doi: 10.1146/annurev.publhealth.25.101802.123134 15015932 10.1146/annurev.publhealth.25.101802.123134

[24] Roter DL, Hall JA, Aoki Y. Physician gender effects in medical communication: a meta-analytic review. *JAMA* 2002;288(6):756–764. 12169083 10.1001/jama.288.6.756

[25] Tsugawa Y, Jena AB, Figueroa JF, Orav EJ, Blumenthal DM, Jha AK. Comparison of Hospital Mortality and Readmission Rates for Medicare Patients Treated by Male vs Female Physicians. *JAMA Intern Med* 2017;177(2):206–213. doi: 10.1001/jamainternmed.2016.7875 27992617 10.1001/jamainternmed.2016.7875 PMC5558155

[26] Ceci S, Williams W. Understanding current causes of women's underrepresentation in science. *Proc Natl Acad Sci USA* 2011;108:3157–62. doi: 10.1073/pnas.1014871108 21300892 10.1073/pnas.1014871108 PMC3044353

[27] Lempp H, Seale C. Medical students’ perceptions in relation to ethnicity and gender: a qualitative study. *BMC Med Educ* 2006;6:17 doi: 10.1186/1472-6920-6-17 16524457 10.1186/1472-6920-6-17 PMC1435754

[28] Russo G, Gonçalves L, Craveiro I, Dussault G. Feminization of the medical workforce in low-income settings; findings from surveys in three African capital cities. *Hum Resour Health* 2015;31;13:64. 10.1186/s12960-015-0064-9 PMC4521355 26228911

[29] Constant A, Léger PT. Estimating differences between male and female physician service provision using panel data. *Health Econ* 2008;17(11):1295–1315. doi: 10.1002/hec.1344 18404663 10.1002/hec.1344

[30] Desai T, Ali S, Fang X, Thompson W, Jawa P, Vachharajani T. Equal work for unequal pay: the gender reimbursement gap for healthcare providers in the United States. *Postgrad Med J* 2016;92(1092):571–5. doi: 10.1136/postgradmedj-2016-134094 27528703 10.1136/postgradmedj-2016-134094

[31] Ncayiyana DJ. Feminisation of the South African medical profession—not yet nirvana for gender equity. *South Afr Med J Suid-Afr Tydskr Vir Geneeskd* 2011;101(1):5. 10.7196/samj.4705 21626966

[32] Park J, Minor S, Taylor RA, Vikis E, Poenaru D. Why are women deterred from general surgery? *Am J Surg* 2005;190:141–6. doi: 10.1016/j.amjsurg.2005.04.008 15972188 10.1016/j.amjsurg.2005.04.008

[33] Brown JB, Fluit M, Lent B, Herbert C. Surgical culture in transition: gender matters and generation counts. *Canadian Journal of Surgery* 2013;56(3):153–158. 10.1503/cjs.024011 PMC3672427 23484466

[34] Ahmadiyeh N, Cho NL, Kellogg KC, Lipsitz SR, Moore FD Jr, Ashley SW, et al Career satisfaction of women in surgery: perceptions, factors, and strategies. *J Am Coll Surg* 2010;210:23–30. doi: 10.1016/j.jamcollsurg.2009.08.011 20123327 10.1016/j.jamcollsurg.2009.08.011

[35] Bruce AN, Battista A, Plankey MW, Johnson LB, Marshall MB. Perceptions of gender-based discrimination during surgical training and practice. *Medical Education Online* 2015;20: doi: 10.3402/meo.v20.25923 25652117 10.3402/meo.v20.25923 PMC4317470

[36] Hafferty FW. Beyond curriculum reform: confronting medicine's hidden curriculum. *Acad Med* 1998;73:403–7. 9580717 10.1097/00001888-199804000-00013

[37] Hoover EL. Mentoring women in academic surgery: overcoming institutional barriers to success. *J Natl Med Assoc* 2006;98(9):1542–1545. 17019926 PMC2569703

[38] Buddeberg-Fischer B, Stamm M, Buddeberg C, Bauer G, Häemmig O, Knecht M, et al The impact of gender and parenthood on physicians’ careers—professional and personal situation seven years after graduation. *BMC Health Services Research* 2010;10:40 doi: 10.1186/1472-6963-10-40 20167075 10.1186/1472-6963-10-40 PMC2851709

[39] Travis EL, Doty L, Helitzer D. Sponsorship: a path to the academic medicine C-suite for women faculty? Acad Med. 2013;88(10), 1414–1417. doi: 10.1097/ACM.0b013e3182a35456 23969365 10.1097/ACM.0b013e3182a35456

[40] Zerzan J, Hess R, Schur E, Phillips R, Rigotti N. Making the most of mentors: A guide for mentees. *Acad Med* 2009;84(1):140–144. doi: 10.1097/ACM.0b013e3181906e8f 19116494 10.1097/ACM.0b013e3181906e8f

[41] Bickel J. How men can excel as mentors of women. *Acad Med* 2014;89:1100–2. doi: 10.1097/ACM.0000000000000313 24853197 10.1097/ACM.0000000000000313

[42] Burgess DJ, Joseph A, van Ryn M, Carnes M. Does stereotype threat affect women in academic medicine? *Acad Med* 2012;87:506–12. doi: 10.1097/ACM.0b013e318248f718 22361794 10.1097/ACM.0b013e318248f718 PMC3315611

[43] Arrizabalaga P, Abellana R, Viñas O, Merino A, Ascaso C. Gender inequalities in the medical profession: are there still barriers to women physicians in the 21st century? *Gac Sanit* 2014;28(5):363–8. doi: 10.1016/j.gaceta.2014.03.014 24889702 10.1016/j.gaceta.2014.03.014

[44] Kilminster S, Downes J, Gough B, Murdoch-Eaton D, Roberts T. Women in medicine—is there a problem? A literature review of the changing gender composition, structures and occupational cultures in medicine. *Med Educ* 2007;41(1):39–49. doi: 10.1111/j.1365-2929.2006.02645.x 17209891 10.1111/j.1365-2929.2006.02645.x

