# Supplementary material for: A qualitative study on perceptions of surgical careers in Rwanda: A gender-based approach
Source: PLoS One. 2018 May 10;13(5):e0197290. doi: 10.1371/journal.pone.0197290 (PMC5944995; doi:10.1371/journal.pone.0197290)
Supplement: S1 Table — (PDF) [file pone.0197290.s001.pdf]

Table 1. Main themes

**Critical time points**	**Main Themes**
Developing **interest** in surgery as a specialty	Role models
	Patient case encounters
	Exposure to surgery
**Selecting and sustaining** surgical careers	Social expectations about roles within the family
	Physical and mental challenges
	Professional and personal support
	Finances
