# Supplementary material for: A qualitative study on perceptions of surgical careers in Rwanda: A gender-based approach
Source: PLoS One. 2018 May 10;13(5):e0197290. doi: 10.1371/journal.pone.0197290 (PMC5944995; doi:10.1371/journal.pone.0197290)
Supplement: S2 Table — (PDF) [file pone.0197290.s002.pdf]

Table 2. Supporting quotes about selecting and sustaining surgical careers

Social expectations about roles within the family	Physical and mental challenges
• *"The culture itself is a big bias… When you want to join surgery, you know how demanding it is, but if you like it, you tend to sacrifice other things. You can choose another [specialty] for the sake of family and other social responsibilities" (F6).*<br><br>• *"It's unusual for a woman to do surgery in Rwanda; it is not soft, it is hard and not for a woman" (F5).*<br><br>• *"In Rwanda, they say there are some jobs that are made for males and other jobs made for women, culturally… In sciences, people think it was made for males not for females." (F3).*	• *"[The male colleagues] keep an eye on you, they expect you to get more tired than them" (F3)*<br><br>• *"Some may see blood and get too stressed. Some may say, 'I can't! I can't stand while operating.' Some may say, 'I fear to even see a wound!' Women may be physically, emotionally less strong than men. But me, I am not that way, because I feel that I am strong as men" (F4).*<br><br>• *"We are in Africa. [The husbands] say you can do this, you cannot do this" (F3)*<br><br>• *"The advice that I can give to medical students is to females: do not be afraid to join surgery. surgery is a career like any other career. You can make it, you can do what you want to do. Even women can do it. And yes, there are some challenges, but they can face them and they can overcome them, like standing for so long. I sometimes have swollen, tired feet and you still have to go for academic day, but you can still make it" (F1).*<br><br>• *"Surgery requires brain, requires physical work, [and] working hard. Males… in our culture and all over the world, they are stronger so they have been doing stronger things" (M2).*<br><br>• *"There really is no sex selection criteria for doing surgery. It is all 'mentality-driven'" (M1).*

	• *"Factors that push women away from surgery are in their minds" (M2)*<br><br>• *"What a man can do, a woman can do it" (M3).*<br><br>• *"[Women] think that job for women should be easier, that they don't have to work hard" (M4)*<br><br>• *"It's not evidence-based, but I think females do not like stress. They like easy life, like pediatrics, radiology, something in which they are not stressed and can just sit down" (M4).*
