# Supplementary material for: A qualitative study on perceptions of surgical careers in Rwanda: A gender-based approach
Source: PLoS One. 2018 May 10;13(5):e0197290. doi: 10.1371/journal.pone.0197290 (PMC5944995; doi:10.1371/journal.pone.0197290)
Supplement: S1 Appendix — (PDF) [file pone.0197290.s003.pdf]

**Supplement A.**

*Study Setting*

Rwanda is a low-income country in East Africa,[1] where tremendous recent investment in health care has improved life expectancy and infant/maternal health indicators.[2] In 2012, the Rwandan Ministry of Health partnered with a consortium of US academic institutions to create the Human Resources for Health (HRH) Program to increase the quantity and quality of providers in Rwanda.[3] The number of surgical residents increased from 15 in 2012 to 50 in 2016. Despite such progress, significant challenges in infrastructure, staff, and clinical processes remain for delivering safe, effective, and timely surgical care.[4] It is in this context that Rwanda provides an ideal background for understanding perceptions about entering and building surgical careers amidst growing yet still resource-limited settings.

*Researcher Team*

The research team included a surgeon, two surgical residents, and one medical student- all of whom are female. The primary interviewer (SY) is a US medical student with experience in qualitative methodology. The data collection and analysis process were also managed by a US surgical resident (YL) with training and experience in qualitative methodology. The surgeon (ACC) and surgical resident (GK) who have been living and working in Rwanda provided counsel with contextual insight. Prior to the study, SY worked in Rwanda for 5 months and met with Rwandan surgical residents and surgeons to explain the goals of the study.

*Member Checking*

Contextual validity was sought through sharing preliminary study findings to Rwandan surgeons and residents so that they could respond to and advise on the validity of the emerging themes. The process of “member checking” facilitated a feedback mechanism on the interpretation of our results from individuals who are living and working daily in the context of this study (Creswell 2000).

*Interview Guide Sample Questions*

1. How did you become interested in general surgery? What sparked your initial interest?
2. What were the most important factors in your decision to pursue surgery? Have these factors changed over time?
3. Are there any factors that would have helped assure your selection of surgery?
4. Were you considering any other specialties? Why did you decide to not pursue those alternatives?
5. Have you ever had any reservations/ hesitations about your specialty selection? Why do you think so?
6. Are there any specific aspects of Rwanda that you think contribute to more men than women selecting surgery as a specialty?
7. Is there anything you wished you would have known and considered before committing to surgery as a specialty?
8. Are you planning on doing a fellowship? Have you ever changed your mind about doing a fellowship; why?
9. Do you have any concerns about future job, financial, or personal prospects?
10. What do you think are your strengths or weaknesses as a surgeon?
11. Do you think your strengths or weaknesses are associated with any gender stereotypes?

12. Do you think your gender has ever shaped the professional opportunities you have been interested in during your surgical training?
13. Which support mechanisms are most important to you; why?
14. What does it mean to you to feel well-supported in pursuing a career in surgery?
15. Is there anything you would change about the surgical training process?

*Supplement A. References*


1. The World Bank. Rwanda: Country at a glance [The World Bank web site]. Available at: http:// www.worldbank.org/en/country/rwanda. Accessed April 23, 2017.
2. National Institute of Statistics of Rwanda (NISR) [Rwanda], Ministry of Health (MOH) [Rwanda], and ICF International. 2015. Rwanda Demographic and Health Survey 2014-15: Key Indicators. Rockville, Maryland, USA: NISR, MOH, and ICF International.
3. Binagwaho A, Farmer Paul E, Nsanzimana Sabin, et al. Rwanda 20 years on: investing in life. Lancet 2014;384(9940):371-375.
4. Petroze RT, et al. Comprehensive national analysis of emergency and essential surgical capacity in Rwanda. British Journal of Surgery 2012. 99(3):436-443.
5. Creswell JW, Miller DL. Determining validity in qualitative inquiry. Theory Into Pract. 2000;39:124–130.
